# MeNPs-PEDOT Composite-Based Detection Platforms for Epinephrine and Quercetin

**DOI:** 10.3390/bios14070320

**Published:** 2024-06-25

**Authors:** Sorina Alexandra Leau, Mariana Marin, Ana Maria Toader, Mihai Anastasescu, Cristian Matei, Cecilia Lete, Stelian Lupu

**Affiliations:** 1 Department of Electrochemistry and Corrosion, Institute of Physical Chemistry–Ilie Murgulescu of the Romanian Academy, 202 Splaiul Independentei, 060021 Bucharest, Romania; sorina.leau@upb.ro (S.A.L.);; 2 Department of Analytical Chemistry and Environmental Engineering, Faculty of Chemical Engineering and Biotechnologies, National University of Science and Technology Politehnica of Bucharest, 1-7 Polizu Gheorghe, 011061 Bucharest, Romania; 3 Department of Inorganic Chemistry, Physical Chemistry and Electrochemistry, Faculty of Chemical Engineering and Biotechnologies, National University of Science and Technology Politehnica of Bucharest, 1-7 Polizu Gheorghe, 011061 Bucharest, Romania

**Keywords:** sensing platform, alternating currents, nanocomposite materials, quercetin, epinephrine

## Abstract

The development of low-cost, sensitive, and simple analytical tools for biomolecule detection in health status monitoring is nowadays a growing research topic. Sensing platforms integrating nanocomposite materials as recognition elements in the monitoring of various biomolecules and biomarkers are addressing this challenging objective. Herein, we have developed electrochemical sensing platforms by means of a novel fabrication procedure for biomolecule detection. The platforms are based on commercially available low-cost conductive substrates like glassy carbon and/or screen-printed carbon electrodes selectively functionalized with nanocomposite materials composed of Ag and Au metallic nanoparticles and an organic polymer, poly(3,4-ethylenedioxythiophene). The novel fabrication method made use of alternating currents with controlled amplitude and frequency. The frequency of the applied alternating current was 100 mHz for the polymer deposition, while a frequency value of 50 mHz was used for the in situ electrodeposition of Ag and Au nanoparticles. The selected frequency values ensured the successful preparation of the composite materials. The use of readily available composite materials is intended to produce cost-effective analytical tools. The judicious modification of the organic conductive matrix by various metallic nanoparticles, such as Ag and Au, extends the potential applications of the sensing platform toward a range of biomolecules like quercetin and epinephrine, chosen as benchmark analytes for proof-of-concept antioxidant and neurotransmitter detection. The sensing platforms were tested successfully for quercetin and epinephrine determination on synthetic and real samples. Wide linear response ranges and low limit-of-detection values were obtained for epinephrine and quercetin detection.

## 1. Introduction

The development of simple, cost-effective, and sensitive analytical tools with applications in the quantification of various biomolecules is gaining a growing interest and many studies are being devoted to this societal need [1]. The design of these analytical tools is oriented to the investigation of selective sensing materials that can be easily integrated within sensitive transducers based on electrochemical, optical, and mass change detection strategies. The electrochemical transducers provide easily measurable analytical signals that are straightforwardly related to the analyte concentration [2,3]. Furthermore, the direct connection of the transducer with the sensing element can be easily achieved by using composite materials composed of conducting organic or inorganic components. Thus, the sensing materials can be directly prepared on the transducer surface, producing both disposable and reusable sensing platforms [4]. The use of organic conductive polymers characterized by electrical conductivity similar to semiconductors has underpinned sensor technology by providing a versatile approach for the selective modification of the electrochemical transducers. The high stability, electrical conductivity, and optical and electrochemical properties of conductive polymers have led to their successful application in the fabrication of electrochemical sensing platforms [5,6]. An intrinsic property of organic conductive polymers is their capability to incorporate various species, ranging from inorganic fillers to biological elements like enzymes, by simultaneous inclusion during the polymer preparation or subsequent modification of the deposited organic polymer. The preparation of the polymeric matrix directly onto the transducer surface by electrochemical methods provides several advantages over chemical preparation procedures of polymers as bulk or thin membranes, like good control of the polymer thickness, the simultaneous incorporation of inorganic/organic fillers, and the modulation of the electrical conductivity during the polymer preparation. These challenging objectives have been investigated by various research groups with good results regarding the use of organic polymers. The most investigated ones are poly (3,4-ethylenedioxythiophene), namely, PEDOT, polypyrrole, polyaniline, and their derivatives [7,8,9,10]. In addition, the electrocatalytic and optical properties as well as the good biocompatibility with metal nanoparticles like silver and gold have been explored for the construction of reliable electrochemical sensing platforms.

Quercetin (3,3′,4′,5,7-pentahydroxylflavone; QR) is an antioxidant with anti-inflammatory and antiviral properties and is found in vegetables and fruits [11,12,13]. QR is considered an important nutrient in the human diet. Consequently, various analytical methodologies, including chromatographic and electrochemical ones, have been investigated for QR quantification [14,15,16].

Epinephrine (EPI) is an important neurotransmitter with roles in stress management and serves as medication for the treatment of various conditions like anaphylaxis, asthma, and cardiovascular diseases [17]. In recent decades, the assessment of the EPI level in various biological samples like blood, urine, and cerebrospinal fluid has become a center of interest owing to the sharp increase worldwide in neurological disorders such as schizophrenia, depression, anxiety, insomnia, Parkinson’s, and Alzheimer’s diseases [18]. EPI belongs to the catecholamines and includes a catechol moiety that is exploited as an electrochemical label for its quantification [19,20,21,22].

In this work, sensing platforms were developed by means of the alternating (sinusoidal) currents protocol for the detection of QR and EPI. The sensing materials were composed of silver nanoparticles (AgNPs) and gold nanoparticles (AuNPs) and the low-cost organic polymer PEDOT. The nanocomposite materials were electrodeposited onto disk electrodes made of glassy carbon (GCE) and/or graphite screen-printed carbon electrodes by applying an alternating current of controlled frequency and amplitude. The obtained sensing platforms were tested, with good analytical performance for QR and EPI electroanalysis in real samples with various compositions.

## 2. Materials and Methods

### 2.1. Materials and Instrumentation

The reagents and the chemicals were purchased from Sigma-Aldrich and used as received. Aqueous solutions were made daily with bi-distilled water. The electrochemical experiments were performed using Autolab potentiostat/galvanostats (models 302N and Vionic, Metrohm AG) and the NOVA 2.1.5 software installed on a PC. Disk electrodes (d = 3 mm) made of glassy carbon (Metrohm AG) and/or graphite screen-printed carbon electrodes (Dropsens) served as working electrodes and were modified with the nanocomposite materials. A silver–silver chloride (Metrohm AG) electrode was the reference electrode. A rod of glassy carbon from Metrohm AG was the auxiliary electrode. The measurements and the analytical applications were performed at ambient temperature under an argon atmosphere.

The morphological characterization of the composite materials was carried out by means of atomic force microscopy (AFM) using an XE-100 (Park Systems) instrument equipped with decoupled sample/probe scanners, as recommended for minimizing the tip–sample interaction. The scanning electron microscopy measurements were made with the Tescan Vega3 LMH apparatus.

### 2.2. Preparation of the Sensing Platforms

The sensing platforms were prepared by the SC protocol in two steps using the following parameters: firstly, the PEDOT matrix was generated from 10^−2^ M EDOT in 0.1 M LiClO_4_ solution by using an alternating current with a frequency of 0.1 Hz and an amplitude value of 20 μA, which was applied to a fixed anodic current of 25 μA for 300 s deposition time; secondly, the AgNPs were synthesized from a 5 mM AgNO_3_ precursor in aqueous solution containing 50 mM sodium citrate using an alternating current with a 0.05 Hz frequency, and an amplitude value of 0.25 μA that was applied to a fixed cathodic current of −2 μA, with a 300 s electrodeposition time.

The AuNPs-based sensing platform was prepared by a similar approach. Firstly, the PEDOT matrix was deposited by means of the SC approach. During the optimization process of the PEDOT deposition, another approach was applied that consists in the application of an SC excitation signal with a narrow frequency in the range 100.1 to 100.001 mHz and an amplitude of 25 μA onto an anodic current of 15 μA. The electrodeposition of the polymeric matrix by both SC procedures was performed onto an Au-coated quartz crystal (Inficon, Maxtek) electrode with a 5 MHz frequency and 1.37 cm^2^ active area. The deposited mass was estimated from the variation in the crystal’s frequency during the SC electrodeposition of the material. Subsequently, AuNPs were prepared on the PEDOT matrix from 5 mM HAuCl_4_ in 0.5 M sulfuric acid by applying these parameters: an alternating current with 0.05 Hz frequency, and an amplitude value of 25 μA applied over a cathodic current with a magnitude of −15 μA for 100 s electrodeposition time. The parameters for PEDOT and AuNPs preparation were selected aiming to enhance the analytical characteristics of the sensing platform compared to a previous study related to epinephrine detection [22].

### 2.3. Analytical Applications

The sensing platforms were applied to quercetin and epinephrine quantification in synthetic and real samples. The analytical detection of the target analytes was achieved in a phosphate-buffered system (0.1 M PBS with pH of 7.0) by a cyclic voltammetric technique. Calibration plots were constructed for each analyte by multiple standard addition using external standards. The overall analytical performance of the sensing platforms was evaluated for the target analytes. The real samples analysis was performed after dilution with PBS. The urine sample was used with the informed consent from a healthy volunteer. The urine samples were stored in the refrigerator. Prior to analysis, the urine samples were diluted with buffer system without additional pretreatments. Afterwards, the urine samples were subjected to the analysis. The grape must was purchased from a local store and was kept in refrigerator without any pretreatment. The grape must samples were diluted with buffer system prior to the analysis. The standard addition procedure was used in the analytes quantification in grape must samples for quercetin, and urine samples for epinephrine, respectively.

## 3. Results and Discussion

### 3.1. Preparation and Characterization of the Sensing Platforms

#### 3.1.1. AgNPs-Based Sensing Platform

The optimum electrochemical parameters of the SC method were established in order to achieve an enhanced overall analytical performance of the sensing platforms. In this way, the deposition conditions of the organic polymer matrix from our previous study [22] were optimized for the subsequent AgNPs electrodeposition. The optimum parameters were a constant current of 25 μA, and frequency and amplitude of 100 mHz and 20 μA, respectively, for the alternating current, while the deposition time was 300 s. In the case of AgNPs, the following parameters were investigated for optimization: constant cathodic currents of −2.0 and −10.0 μA; alternating currents with amplitudes of 0.25, 1.5, and 3.5 μA. The frequency of the SC signal was 50 mHz. A constant deposition time of 300 s was used.

**Figure 1:**
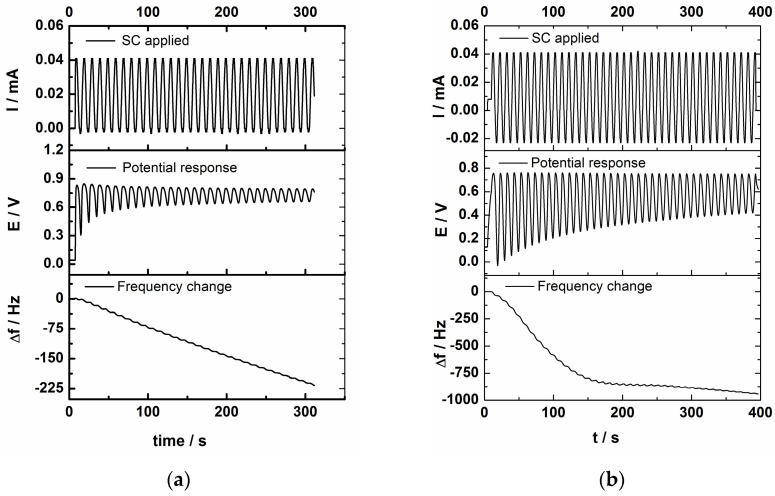
The sinusoidal current, the potential response, and the changes in frequency recorded for the preparation of PEDOT matrix via the SC approach onto gold-covered quartz crystal electrode by using (**a**) fixed frequency of 100 mHz and (**b**) sweeping frequency in the range from 100.1 to 100.001 mHz. Electrolyte solution containing 10^−2^ M EDOT in 0.1 M LiClO_4_.

The PEDOT coating was prepared by using both a fixed-frequency SC signal as well as by sweeping the frequency of the SC signal in a narrow value range, as described in the experimental section. The deposition of PEDOT onto a gold-coated quartz crystal electrode (Maxtek) was achieved with a frequency of 5 MHz and 1.37 cm^2^ active area of the electrode. The changes in the crystal resonator frequency (Δf) were recorded simultaneously with the applied SC signal and the potential response using an I/O card and the experimental setup that includes the electrochemical quartz crystal microbalance (Maxtek). It should be noted that the SC preparation procedure can be performed by means of an FRA module, but without the I/O card, with the same results. Figure 1 displays the sinusoidal current, the system response, and the changes in the frequency during the PEDOT matrix preparation.

The SC signal generates a sinusoidal potential ranging between 0.3 and 0.85 V that ensures the electrodeposition of the polymer matrix (see Figure 1a). The sinusoidal shape of all the recorded signals, i.e., the applied excitation SC, the potential response, and the frequency change in the Au-coated crystal, can be noted. The polymerization process occurs on the application of the positive (anodic) part of the alternating current cycle, and this is also corroborated with the change in the frequency of the crystal resonator, which shows mass deposition onto the quartz crystal electrode. At the end of each SC cycle, there is an increase in the frequency of the crystal due to the expulsion of the counterions and/or the solvent molecules. This behavior is consistent with the peculiarities of the electrodeposition of conducting polymers. The electro-gravimetric measurements provide additional information on the characteristics of the SC approach. The mass of PEDOT deposited onto the electrode was estimated according to the Sauerbrey equation and assuming that a rigid deposit was obtained [23]:

Δf = −(2 f_0_^2^ Δm)/(A ρ^1/2^ μ^1/2^), (1)

where f_0_ represents the resonant frequency, Δm stands for the mass change, A is the active area (1.37 cm^2^), ρ refers to the quartz density (2.648 g/cm^3^), and μ represents the shear modulus of quartz (2.947 × 10^11^ g/cm s^2^). Thus, a deposited mass of 5.2 (±0.3) μg/cm^2^ was obtained for PEDOT. A theoretical estimation of the PEDOT amount deposited onto the electrode surface could be made using the electrical charge and the Faraday equation considering 2.25 electrons per molecule and assuming a 100% efficiency of the polymerization process. Thus, a value of 3.1 μg/cm^2^ could be obtained. The difference may be due to the fact that the theoretical calculation does not take into account the counterions and the solvent molecules that are incorporated/expelled from the polymer layer. However, the obtained results correlate well and confirm the suitability of the SC approach in PEDOT deposition.

In the case of the SC procedure, based on scanning of the frequency in the range from 100.1 to 100.001 mHz there was a larger change in the crystal resonator frequency in the first 3 min of the polymerization process, followed by a smaller slope of the crystal frequency change (see Figure 1b). The variation in the frequency Δf is like that of the SC procedure based on a fixed frequency of the applied sinusoidal current, attesting that a similar mechanism is involved in the polymerization process. The potential response is in the range from 0.25 to 0.75 V. However, the deposition time is not easily controlled, resulting in larger deposition times, of ca. 400 s, than those theoretically calculated (300 s) taking into account the number of the scanned frequencies and the integration cycles. In this case, a mass change of 22.4 (±7.2) μg/cm^2^ was obtained. Taking into consideration that the deposition time should be precisely controlled during the polymerization process in order to ensure higher reproducibility in the preparation of the sensing platforms, even if a higher amount of the deposited polymer is obtained, the fixed-frequency-based SC approach was further applied in the preparation of the sensing platforms.

The in situ electrodeposition of the Ag and Au nanoparticles was carried out by the SC procedure in order to prepare the corresponding PEDOT-AgNPs and PEDOT-AuNPs sensing platforms on various substrates. The SC procedure was applied by means of the FRA module using the experimental parameters described in the experimental section. Actually, the SC method requires only the FRA module without the EQCM and I/O card. As an example of the use of the I/O card only, the in situ electrodeposition of the AuNPs onto the PEDOT matrix is presented in Figure S1 (see Supplementary File). Thus, the amount of AuNPs could be estimated from the electrical charge and using the Faraday equation assuming a 100% efficiency of the electrodeposition process. An amount of electrodeposited AuNPs of 3.4 μg/cm^2^ was obtained. The amount of electrodeposited AgNPs was also estimated and a value of 3.7 μg/cm^2^ was obtained.

After the optimization of the deposition procedures, the morphology of the PEDOT-AgNPs and PEDOT-AuNPs composite materials was investigated.

**Figure 2:**
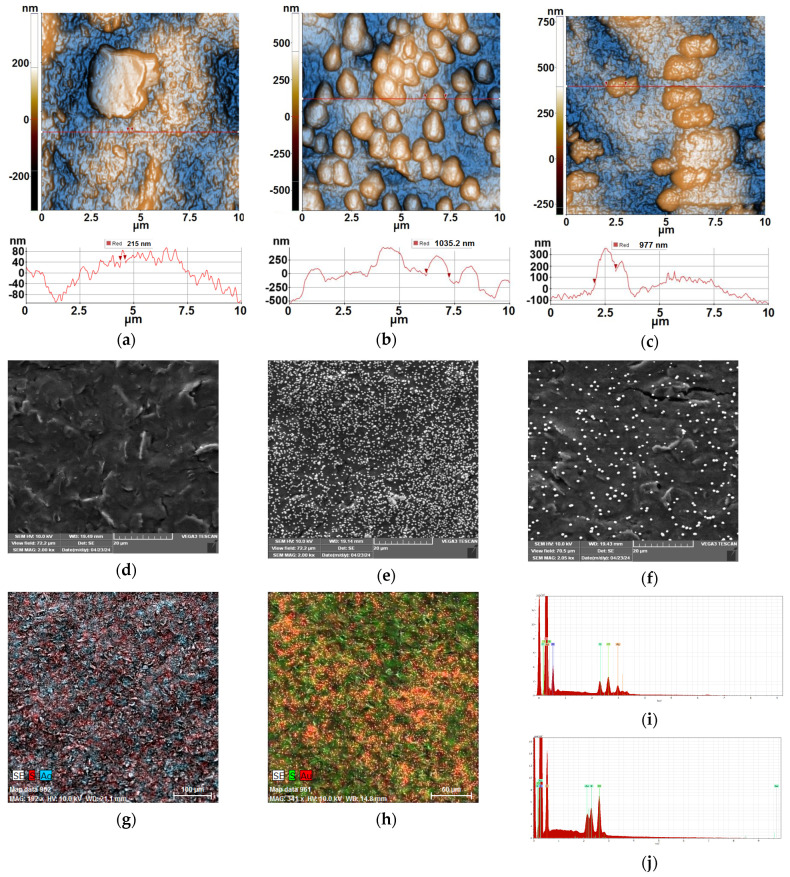
Two-dimensional enhanced-contrast topographic AFM images (with characteristic line scans) scanned over 10 µm × 10 µm of (**a**) PEDOT, (**b**) PEDOT-AgNPs, (**c**) PEDOT-AuNPs; SEM images of (**d**) PEDOT, (**e**) PEDOT-AgNPs, and (**f**) PEDOT-AuNPs. EDX elemental mapping and spectra for (**g**,**i**) PEDOT-AgNPs, and (**h**,**j**) PEDOT-AuNPs.

Figure 2 presents the bi-dimensional (2D) enhanced-contrast AFM images, topographic mode, for the PEDOT, PEDOT-AgNPs, and PEDOT-AuNPs samples registered at the scale of 10 µm × 10 µm, accompanied by characteristic line scans (with selected particles indicated by red arrows), emphasizing the nanostructured morphology of the investigated samples, and the SEM images of PEDOT, PEDOT-AgNPs, PEDOT-AuNPs.

Figure 2a shows the topography of the PEDOT film electrochemically deposited on a screen-printed carbon electrode surface. It can be observed that the PEDOT matrix exhibits a morphology consisting of small quasi-hemispherical nanoparticles with a mean diameter of ~100 nm (the particles have diameters from tens of nm up to ~150 nm). The RMS roughness is 93 nm and the peak-to-valley is ~694 nm but these values are strongly influenced by the topographical irregularities (roughness parameters) of the carbon substrate. Figure 2b represents the morphology of the AgNPs grown on the previously deposited PEDOT matrix. It can be remarked that the AgNPs are grouped in forms of slightly textured balls (lumps) of around 1 µm in diameter which are relatively uniform distributed over the surface of the PEDOT-AgNPs sample. Due to the presence of the AgNPs, there is a clear increase in the roughness parameters, so that the Rq is around 224 nm and the peak-to-valley is ~1.34 µm (the largest values in the series). The roughness parameters of the PEDOT-AuNPs sample are Rq~167 nm and Rpv~1.19 µm (see Figure 2c). The SEM images show the increased roughness of PEDOT (Figure 2d) and the homogeneous distribution of the AgNPs (Figure 2e) and AuNPs (Figure 2f), respectively. The EDX elemental mapping of the PEDOT-AgNPs (Figure 2g) and PEDOT-AuNPs (Figure 2h) materials shows a relatively homogeneous distribution of the Ag and Au nanoparticles in the composite materials. The presence of the metallic nanoparticles is demonstrated by both AFM and SEM (Figure 2i,j) measurements and the increased roughness (suggesting an increased surface area) of the composite materials could improve the sensibility of the sensing platforms.

**Figure 3:**
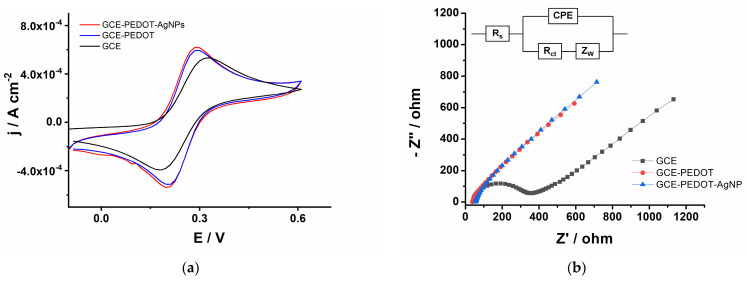
(**a**) CV traces registered at unmodified GCE, PEDOT, and PEDOT-AgNPs at 50 mV/s potential scanning rate in 5 mM Na_4_[Fe(CN)_6_], with 0.5 M KNO_3_ as supporting electrolyte. (**b**) The corresponding EIS spectra of bare GCE, PEDOT, and PEDOT-AgNPs recorded in 5 mM Na_4_[Fe(CN)_6_]/K_3_[Fe(CN)_6_], with 0.5 M KNO_3_ as supporting electrolyte. Inset: the equivalent electrical circuit used in the fitting of the experimental data.

The performance of the AgNPs-PEDOT platform was evaluated by cyclic voltammetry (CV) in a solution of 5 mM Na_4_[Fe(CN)_6_] (see Figure 3a). The peak of the anodic current increases for the PEDOT-AgNPs-modified electrode compared to the GCE by a factor of 1.5, illustrating the improved electron transfer process of the nanocomposite material. The current is still higher than that for the polymer-only matrix due to the presence of the silver nanoparticles embedded within the organic layer. The significant contribution of the polymeric matrix is thanks to the higher porosity of the polymer endowed by the SC method. The electrochemical properties were studied by using the impedance spectroscopy technique (EIS) (see Figure 3b). All EIS measurements were performed in a solution of Na_4_[Fe(CN)_6_]/K_3_[Fe(CN)_6_] in equal concentrations at 5 mM in a 0.5 M KNO_3_ supporting electrolyte. The EIS spectra were recorded at the open circuit potential value in the frequency range from 10 kHz to 0.05 Hz using an excitation signal with an amplitude of 5 mV (rms). The spectrum of the GCE is dominated by a large semicircle in the high-frequency region, which is determined by the charge transfer resistance. The charge transfer resistance can be estimated from the diameter of the semicircle in the high-frequency part of the spectra. Compared to the GCE, the PEDOT and PEDOT-AgNPs displayed lower charge transfer resistance values, and this is attributed to the electrocatalytic properties of the coatings and to their higher porosity. The imaginary part of the impedance (Z″) in the EIS spectra is related to the capacitive properties of the electrode. For the PEDOT-AgNPs and PEDOT coatings, the imaginary parts of the impedance are similar and suggest that the inclusion of Ag nanoparticles in the PEDOT matrix does not significantly increase the redox capacitance of the composite coating. Usually, an increase in the capacitance due to the modifying layer could reduce the sensibility of the analytical measurements by increasing the background current. From this point of view, the inclusion of AgNPs within the PEDOT layer does not significantly increase the capacitance and this behavior could bring benefits in the analytical applications. The EIS spectra were fitted with a Randles equivalent electrical circuit (the inset of Figure 3b), which consists of the solution resistance (R_s_), the charge transfer resistance (R_ct_), the constant phase element (CPE), and the infinite Warburg diffusion element (Z_W_). The charge transfer resistance values for the PEDOT-AgNPs, PEDOT-AuNPs, PEDOT, and the unmodified GCE were as follows, in the same order: 179.2 Ω (chi squared, 3.7 × 10^−4^; sum of squares, 0.009); 38.1 Ω (chi squared 1.1 × 10^−4^; sum of squares, 0.003); 215.9 Ω (chi squared 3.4 × 10^−4^; sum of squares, 0.008); 270.0 Ω (chi squared 1.1 × 10^−3^; sum of squares, 0.04). The charge transfer resistance decreases for the AuNPs- and AgNPs-based platforms compared to the PEDOT-modified electrode and the unmodified GC electrode. These results point to an enhancement in the electron transfer rate at the electrode/solution interface due to the presence of the Au- and Ag-based composite materials. Consequently, the metallic nanoparticles improve the electrical conductivity and the electron transfer capability of the corresponding electrochemical sensing platforms.

The *Ip* versus *v*^1/2^ dependence was used in assessing the electroactive surface area (ESA) of the AgNPs platform for various experimental parameters of the SC method. The highest ESA value of 0.132 cm^2^ was obtained for the optimum electrochemical parameters described in Section 2.2. Compared to the unmodified GC electrode, a roughness factor of 1.9 was obtained for the AgNPs-based sensing platform, attesting to the enhanced electron transfer properties. Thus, the data obtained by CV well corroborated the EIS measurements.

**Figure 4:**
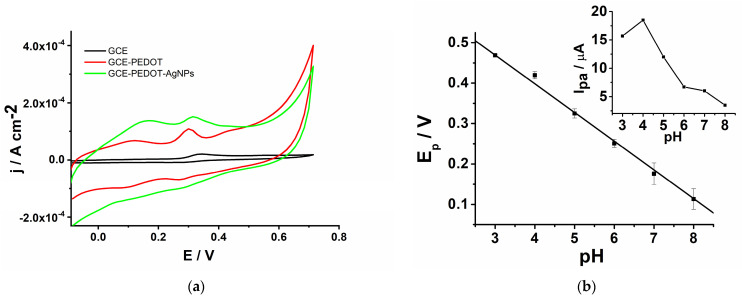
(**a**) CV registered with GCE, PEDOT, and PEDOT-AgNPs sensing platform in acetate buffer system (pH = 5) with 40 μM QR, with a 50 mV/s potential sweep rate. (**b**) Influence of the pH on the QR detection of the PEDOT-AgNPs sensing platform in buffered systems with pH ranging from 3 to 8 containing 50 μM QR. Potential sweep rate: 50 mV/s.

The electrochemical behavior of QR was studied at both modified and unmodified electrodes, aiming to investigate the electrocatalytic properties of the composite sensing material and the obtained CV data are illustrated in Figure 4a. The QR oxidation peak potential for the PEDOT-AgNPs sensing material was +0.31 V, lower than that of the unmodified GCE (+0.36 V) and similar to that observed for PEDOT (0.31 V). In addition, a higher anodic peak current at the PEDOT-AgNPs-based sensor was observed. The results illustrate the good electrocatalytic activity of the composite material, suggesting it could be applied in the fabrication of analytical devices for QR electroanalysis.

A pH study related to the QR behavior of the sensing platform was performed (see Figure 4b). Higher anodic currents for QR oxidation were obtained at pH values of 4 and 5. The peak potential related to QR oxidation is shifted toward less positive values when the pH increases from 3 to 8. The corresponding equation for the linear regression is *Ep* (V) = 0.68 − 0.070 pH; r = 0.9967. A slope with a value of −70 mV/pH unit was obtained and this suggests an equal number of protons and electrons related to the oxidation of the analyte [24,25]. Therefore, the analytical applications and the subsequent investigation of QR were performed in a buffered solution with pH = 5.0.

The oxidation of QR is a process controlled by both diffusion and adsorption according to the dependency of the peak current versus the potential sweep rate (see Figure S2). The equation of the peak potential dependence versus the logarithm of the potential sweep rate was as follows: *E_p_* (V) = 0.028 *ln υ* + 0.395. The estimated electron number was *n* = 1.8, considering a transfer coefficient α = 0.5, suggesting a two-electron process [24].

The electrochemical oxidation of QR was also estimated by electronic structure calculations. The calculations were performed with the Gaussian09 code [26] using the B3LYP functional and 6-311+G basis set [27]. The geometry optimization of quercetin was initiated starting from the experimental structure reported (as crystallographic information file, cif) in Ref. [28].

**Scheme 1:**
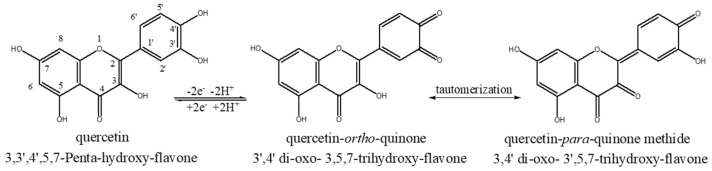
The structure of quercetin and its oxidized tris-ketone forms.

The electronic structure calculations were used as numeric experiments, checking possible scenarios of quercetin oxidation. The mildest step of oxidation would be the transformation of a phenol group into a ketone. Overall, the formation of a ketone must be a two-electron process (see Scheme 1).

On the left-hand side of Scheme 1, one may see the standard labeling of carbon atoms in the flavone skeleton of QR. In principle, formulating the oxidation as the removal of two hydrogen atoms from the hydroxy groups of QR, there exist ten combinatorial possibilities to choose two hydrogen atoms out of the five OH groups. However, only two species from this formal recipe can be drawn as closed Lewis-type structures, shown in the middle and on the right-hand side of Scheme 1. The other eight possibilities are frustrated from the point of view of satisfied valency. On the other hand, all the ten dehydrogenated species can be computed, as hypothetical structures, with three ketone groups, one pre-existing at carbon atom #4 and two resulting from eliminating hydrogens from hydroxyl groups at #*i* and #*j* carbons. Thus, labeling the resulting tris-ketone by the couple of #*i*–#*j* labels, the following series of ten imaginable oxidation products can be obtained, {3′-4′, 3-4′, 7-4′, 3-6, 3-3′, 5-3′, 7-3, 3-5, 5-3′, 5-7}, having the respective relative energies {0.0, 5.2, 25.0, 28.8, 32.0, 36.4, 40.7, 40.9, 51.6, 57.5}, with all values in kcal/mol. One observes that the most stable structures are those formulated as *ortho*-quinones, with dehydrogenation at the former 3′-4′ pair of hydroxy groups, followed closely, by ca. 5 kcal/mol, by the 3-4′ structure, with a *para*-quinone methide pattern, namely, the formulas sketched in Scheme 1.

#### 3.1.2. AuNPs-Based Sensing Platform

The AuNPs sensing platform was developed by the optimization of the SC deposition parameters compared to our previous study [22]. The deposition of the PEDOT matrix onto the Au-coated quartz crystal electrode was achieved by microgravimetric measurements, as described in Section 3.1.1. The change in the frequency of the crystal was recorded together with the applied SC and the potential response. After the PEDOT deposition, the AuNPs were electrogenerated by means of the SC procedure. The AuNP electrodeposition is sensitive to the electrochemical parameters. Thus, the cathodic constant current as well the amplitude of the alternating current were further optimized. Therefore, the AuNPs deposition was achieved using an alternating current characterized by a frequency of 50 mHz and an amplitude of 25 μA that was applied onto a constant cathodic current with a magnitude of −15 μA for an electrodeposition time of 100 s. These experimental parameters allowed the successful preparation of AuNPs on a PEDOT matrix characterized by a high roughness and homogeneous distribution of Au nanoparticles, as demonstrated by the AFM and SEM measurements described above.

The AuNPs-based platform was studied by the CV technique (see Figure S3a,b). A higher anodic current was observed for the PEDOT-AuNPs sensing platform compared to the bare GC electrode. Also, the peak potential difference was smaller for the AuNPs-based sensing platform. These results suggest the improved electron transfer characteristics of the AuNPs-based composite material. The anodic peak current of the AuNPs sensing platform is similar to that obtained at the PEDOT-modified electrode, confirming that the deposition of AuNPs does not increase the capacitance of the modifying layer nor impede the roughness of the underlying PEDOT matrix. The roughness factor compared to the unmodified electrode is 1.5 for the AuNPs sensing platform, which provides an enhanced analytical sensitivity of the measurements. The process of epinephrine oxidation was studied using the PEDOT-AuNPs sensing platform at various potential scan rates (see Figure S3c). The peak potential for EPI oxidation increases with the potential sweep rate. The electron number related to the oxidation of epinephrine could be computed from the potential sweep rate study, and a value of *n* = 2 was obtained.

### 3.2. Analytical Applications of the Sensing Platforms

#### 3.2.1. Determination of Quercetin Using AgNPs-Based Sensing Platform

**Figure 5:**
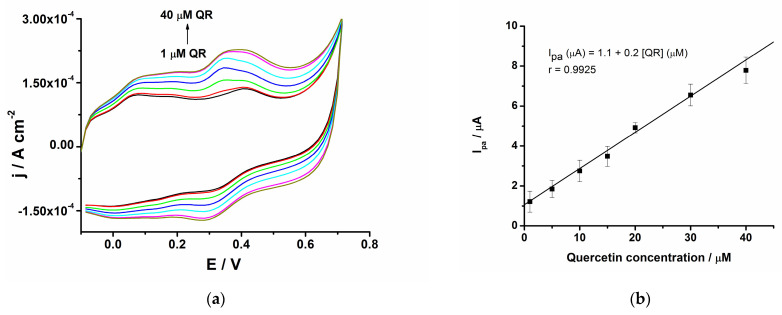
(**a**) CVs for the PEDOT-AgNPs sensing platform in acetate buffer system (pH = 5) in the presence of various amounts of QR: 1, 5, 10, 15, 20, 30, and 40 μM. Potential sweep rate: 50 mV/s. (**b**) The calibration curve of the AgNPs platform.

Based on the previous results related to the electrochemical characterization, electroanalysis of QR by the sensing platform was performed. The QR electroanalysis was performed in acetate buffer with optimum pH by means of the cyclic voltammetry technique. The CV traces recorded using the PEDOT-AgNPs sensing platform in solution containing QR at various concentrations are illustrated in Figure 5. The AgNPs platform exhibited an increase in the anodic current that was linear with the analyte concentration from 1 to 40 μM, with the following equation of regression: *I* (µA) = 1.1 + 0.2 [*QR*] µM (*r* = 0.9925). The sensitivity of the sensing platform was calculated as the ratio between the calibration plot slope and the electrode surface area. The obtained sensitivity was 2.8 μA μM^−1^ cm^−2^. The limit of detection (LOD) and the limit of quantification (LOQ) were assessed by using the formula *LOD* = 3 *α/m* and *LOQ* = 10 *α/m*, where *α* is the standard error of the regression, while *m* represents the slope obtained from the calibration curve. The LOD and LOQ values were 2.8 and 9.5 μM, respectively. The obtained values highlight the analytical performance of the AgNPs platform in the electroanalysis of QR in buffered systems.

The repeatability and reproducibility of the AgNPs-based platform were estimated in terms of relative standard deviation (RSD%). A repeatability value of 3.3% was obtained by measuring QR at a 40 μM level three times with the same sensing platform. The reproducibility was equal to 6.1% for the same QR level using three sensing platforms developed under the same experimental conditions. The obtained values underline the good performance of the SC preparation procedure and the suitability of the AgNPs sensing platform for practical applications.

**Figure 6:**
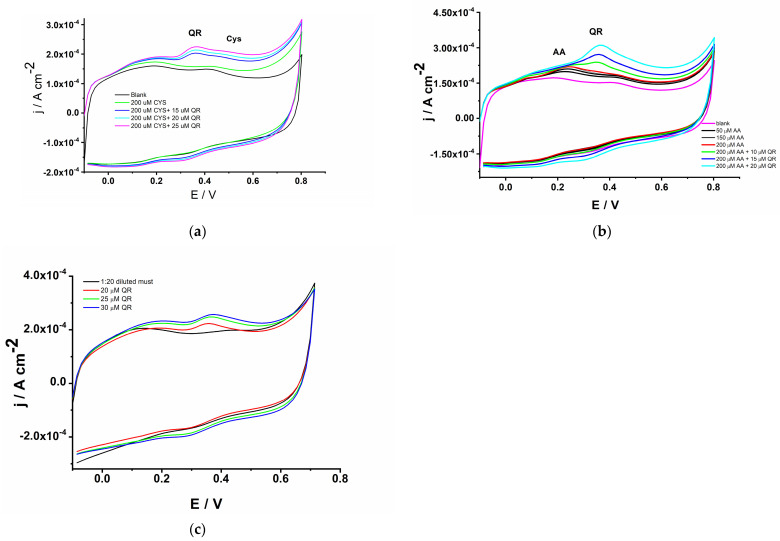
CVs for the PEDOT-AgNPs platform in acetate buffer system (pH = 5) containing QR and various amounts of (**a**) cysteine and (**b**) ascorbic acid. (**c**) The response of the PEDOT-AgNPs platform in diluted grape must sample spiked with 20, 25, and 30 μM QR in acetate buffer system (pH = 5), with a 50 mV/s potential scanning rate.

The selectivity of the developed PEDOT-AgNPs platform was evaluated using as major interfering molecules cysteine (CYS) and ascorbic acid (AA). Their influence on the analytical response was assessed by CV (see Figure 6). The separation of peak potentials for QR and the interfering species CYS and AA were 80 and 150 mV, respectively. The obtained peak potential differences ensure QR detection without major interference. Also, the change in the anodic peak current for QR oxidation is ca. 8% in the presence of various amounts of interfering species, demonstrating that QR quantification is not affected substantially.

The sensing platform was also applied to QR determination in real samples. The grape must samples were diluted with acetate buffer at a 1:20 ratio and the analytical signal was recorded. Furthermore, the real samples were spiked with known amounts of QR. The corresponding CV traces recorded using the AgNPs-based sensing platform for the grape must sample are illustrated in Figure 6c. The peak current of the anodic wave located at 0.34 V increases linearly with QR additions, attesting to the performance of the AgNPs platform to respond to the analyte added in the real sample. The resulting linear regression equation for the QR sensing in grape must was as follows: *I* (μA) = 11.28 + 0.23 [*QR*] (µM) (*r* = 0.9922). Considering the obtained sensitivity values, both in the buffer solution and the grape must sample, it can be stated that the matrix effect is very low. Due to the complex composition of grape must in terms of the presence of various antioxidants, it is possible to assess the performance of the sensing platform in QR detection. Thus, the AgNPs-based platform can be used in QR detection in complex samples without a significant matrix effect. The obtained recovery values for grape must samples ranged from 96.46% to 107.27% (see Table 1). The obtained results show the possibility of performing the analysis of real samples using the developed AgNPs platform.

#### 3.2.2. Determination of Epinephrine Using AuNPs-Based Sensing Platform

**Figure 7:**
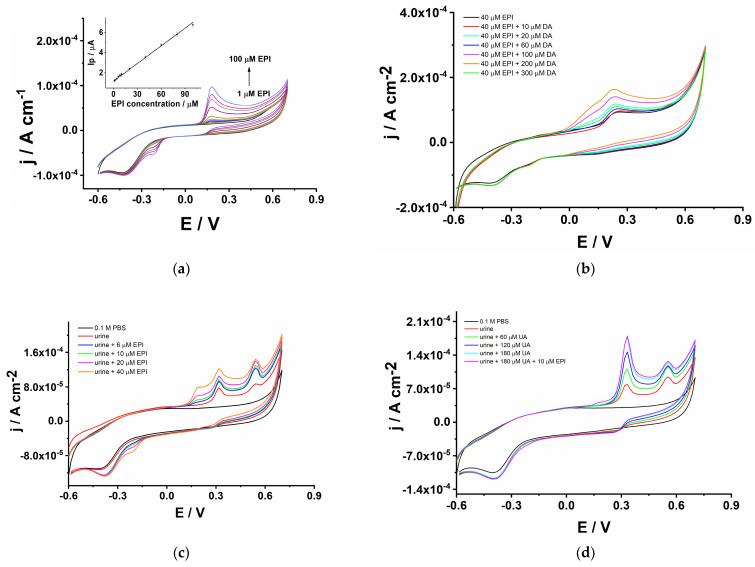
(**a**) Analytical response of the PEDOT-AuNPs sensing platform in phosphate buffer (pH = 7) containing various amounts of EPI (1, 2, 4, 6, 8, 10, 20, 40, 60, 80, and 100 μM); 50 mV/s potential scan rate. Inset: the corresponding calibration plot. (**b**) Analytical response of the PEDOT-AuNPs platform in phosphate buffer (pH = 7) containing 40 μM EPI and various amounts of DA: 10, 20, 60, 100, 200, and 300 μM. (**c**) Analytical response of the AuNPs-based platform in urine sample spiked with various amounts of EPI: 6, 10, 20, and 40 μM. (**d**) Analytical response of the AuNPs-based sensing platform in urine sample spiked with various amounts of UA of 60, 120, 180 μM, and 10 μM EPI, with a 50 mV/s potential scan rate.

The AuNPs-based sensing platform was applied to epinephrine determination in buffered solution with pH = 7.0, which is the optimum value and is close to that of physiological media. The detection mode applied was based on cyclic voltammetry with the standard addition protocol for the construction of the calibration plot. The analytical response of the sensing platform toward EPI was linear from 1 to 100 μM, with the following equation: *Ipa* (μA) = 1.2 + 0.06 [*EPI*] (μM), *r* = 0.9987 (see Figure 7a). The sensitivity of the platform was calculated as the ratio between the calibration curve slope and the electrode surface area. The estimated sensitivity value was 0.85 μA μM^−1^ cm^−2^. From the calibration curve, the obtained detection and quantification limits were as follows: 0.5 μM and 1.7 μM, respectively. These values are better than those previously reported [22].

The repeatability and the reproducibility were measured and expressed as relative standard deviation (RSD%). The repeatability was estimated by measuring an EPI level of 40 μM three times with the same sensing platform and a value of 4.6% was obtained. The use of three different sensing platforms prepared in the same way to measure 40 μM EPI resulted in a reproducibility value of 5.1%. The estimated repeatability and reproducibility for 100 μM EPI were 3.3% and 6.5%, respectively. These values suggest that the sensing platform displays good analytical performance toward EPI determination.

The AuNPs-based platform was also tested for EPI detection in synthetic samples containing dopamine (DA) as well in real samples. The response of the sensing platform to EPI in the presence of DA is displayed in Figure 7b. The anodic wave related to EPI oxidation at 40 μM level is clearly visible at ca. 0.24 V and the anodic wave located at ca. 0.11 V, which is due to DA oxidation, increases linearly with the concentration in the range 10–300 μM DA. A peak potential difference between EPI and DA of 130 mV was observed and this ensures the determination of EPI without a major interference from DA. The sensing platform was applied in a recovery study of EPI in a urine sample. Known amounts of EPI were spiked in the urine sample and the analytical response is displayed in Figure 7c. There are two anodic waves, one located at 0.32 V and the other one at 0.54 V. The addition of known amounts of EPI in the urine sample produces an anodic wave with a peak potential of 0.19 V, which is characteristic of EPI. The anodic peak current of this wave increases linearly with EPI concentration, with a slope of 0.069 μA/μM, which is close to that obtained in buffered solution. This finding demonstrates that the sensing platform can detect EPI in a sample with complex composition without a significant matrix effect. Furthermore, the response of the sensing platform was recorded for a urine sample enriched with uric acid (UA), aiming to investigate the analysis of EPI with the AuNPs platform in samples with complex composition (see Figure 7d). The spiking of the urine sample with UA produces an enhancement in the height of the wave at 0.32 V, attesting that this anodic wave is due to UA. The addition of EPI at the 10 μM level produces the characteristic anodic wave of EPI, even in the real sample and in the presence of a large amount of UA. The recovery values for EPI determination in the urine sample ranged from 104.7 to 108.7% and showed the good capability of the platform in real-sample analysis.

The analytical performance of the developed sensing platforms toward quercetin and epinephrine was compared to that of other sensors discussed previously in the literature (see Table 2). It is seen that the analytical characteristics of the sensing platforms are at the same level as those of other sensors or analytical methodologies. Finally, the developed AgNPs- and AuNPs-based sensing platforms demonstrated good analytical performance in the quercetin and epinephrine determination in real samples.

PrVO_4_@g-CN: praseodymium vanadate@graphitic carbon nitride; CWO/SPCE: cobalt tungstate deposited on screen-printed carbon electrode; PFO/RGO: praseodymium ferrite nanoparticle-decorated reduced graphene oxide composite; ZnO/CNS/CPE: zinc oxide/carbon nanosheet composite-modified carbon paste electrode; Fe@Fe_2_O_3_/AuNPs/N-ZnO: iron-iron(III) oxide/gold nanoparticles/nitrogen doped zinc oxide; ITO: indium tin oxide; ACS: acetate buffer solution; AgNPs: silver nanoparticles; AuNPs: gold nanoparticles; PDA: polydopamine; CeO_2_: cerium oxide nanoparticles; CNFs: carbon nanofibers; CB-PVA: a conductive ink made of carbon black and poly (vinyl acetate) glue; Pd: palladium nanoparticles; Co-Nd: cobalt neodymium bimetallic nanoparticles; Al_2_O_3_: alumina nanoparticles; fMWCNTs: functionalized multiwalled carbon nanotubes; NiO: nickel oxide nanoparticles; PBS: phosphate-buffered solution.

## 4. Conclusions

In this work, sensing platforms based on silver and gold nanoparticle composite materials were investigated. The novel preparation SC procedure endowed the developed AgNPs- and AuNPs-based sensing platforms with good analytical performance toward the target analytes quercetin and epinephrine. The in situ combination of the SC procedure with microgravimetric measurements confirmed the performance of the SC approach in the successful synthesis of the composite materials and provided the amounts of deposited materials. The morphological characterization of the sensing materials underlined the enhanced roughness of the materials and the distribution of the Ag and Au nanoparticles in a homogeneous manner. The PEDOT-AgNPs-based sensing platform showed good overall performance in the QR analysis, with a low LOD and LOQ of 2.8 μM and 9.5 μM, respectively. The AgNPs-based sensing platform was successfully applied in real-sample analysis of QR with good recovery values. The AuNPs-based sensing platform was used in EPI detection and displayed low LOD and LOQ values of 0.5 μM and 1.7 μM, respectively. The AuNPs-based platform showed good selectivity and accuracy for real-sample analysis. The obtained results point to the potential use of the SC method in the fabrication of low-cost and sensitive sensing platforms with applications in antioxidant and neurotransmitter detection.

## Figures and Tables

**Table 1:** Determination of quercetin in grape must sample using PEDOT-AgNPs platform.

Sample	Added (µM)	Found (µM)	Recovery Value (%)	RSD (%)
Grape must sample	20	21.36	106.81	6.48
	25	26.81	107.27	3.53
	30	28.93	96.46	8.44

**Table 2:** A comparison of the analytical performance of different electrochemical sensors applied in quercetin and epinephrine detection.

Electrochemical Sensor	Detection Technique	Limit of Detection (µM)	Linear Response Range (µM)	Sensitivity (µA µM^−1^)	Sample (Matrix)	Ref.
**Quercetin**						
PrVO_4_@g-CN	DPV	0.002	0.05–252	-	0.1 M PBS (pH = 7.1)	[16]
CWO/SPCE	DPV	0.022	0.02–521	0.135	0.1 M PBS (pH = 7)	[24]
PFO/RGO/GCE	DPV	0.026	0.01–257	-	0.1 M PBS (pH = 7)	[25]
ZnO/CNS/CPE	DPV	0.04	0.17–3.63	-	0.1 M PBS (pH = 7)	[29]
Fe@Fe_2_O_3_/AuNPs/N-ZnO/ITO	CV	59	10–1000	-	0.1 M PBS (pH = 7)	[30]
PEDOT-AgNPs	CV	2.8	1–40	0.20	0.1 M ABS (pH = 5)	This work
**Epinephrine**						
GCE-PEDOT-AuNPs	CV	1.4	10–640	2.1 × 10^−2^	0.1 M PBS (pH = 7)	[22]
PDA@CeO_2_/GCE	DPV	8.37	10–200	-	0.1 M PBS (pH = 7.4)	[31]
GCE-CNF-AuNPs	SWV	1.7	50–1000	-	0.1 M PBS (pH = 6)	[32]
Pd/CB-PVA	DPV	0.051	0.75–100	5.48 × 10^−3^	0.1 M PBS (pH = 6.5)	[33]
Co-Nd/Al_2_O_3_@fMWCNTs/GCE	DPV	0.015	0.2–4000	3.23 × 10^−3^	0.1 M PBS (pH = 7)	[34]
GCE/PANI/NiO	SWV	0.05	47–354	6.3 × 10^−3^	0.1 M PBS (pH = 7)	[35]
PEDOT-AuNPs	CV	0.5	1–100	6.0 × 10^−2^	0.1 M PBS (pH = 7)	This work

## Data Availability

The data are contained within the article.

## Supplementary Materials

The following supporting information can be downloaded at: https://www.mdpi.com/article/10.3390/bios14070320/s1, Figure S1: the preparation of AuNPs on GCE-PEDOT; Figure S2: the potential scan rate study of QR at the PEDOT-AgNPs platform; Figure S3: the electrochemical characterization of the PEDOT-AuNPs platform and the analytical response towards EPI.
