# Supplementary material for: MeNPs-PEDOT Composite-Based Detection Platforms for Epinephrine and Quercetin
Source: Biosensors (Basel). 2024 Jun 25;14(7):320. doi: 10.3390/bios14070320 (PMC11274438; doi:10.3390/bios14070320)
Supplement: Supplementary file 1 [file biosensors-14-00320-s001.zip › biosensors-3039911-supplementary.pdf]

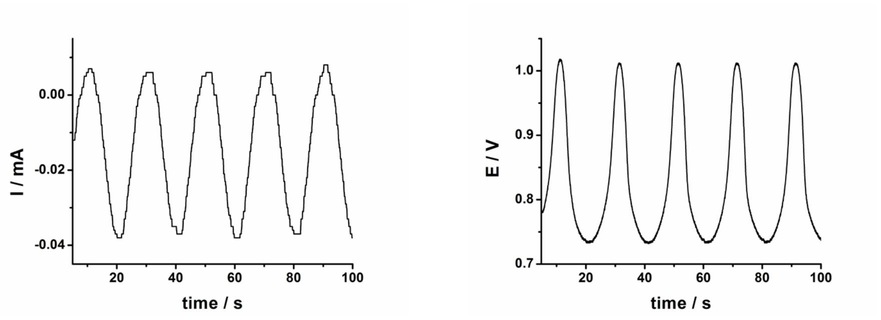

(**a**) (**b**)

Figure S1. (a) The applied SC signal and **(b)** the potential response recorded during the preparation of AuNPs on PEDOT matrix via SC method using an SC with fixed frequency of 50 mHz and amplitude of 25 μA superimposed on a cathodic current of –15 μA, with a deposition time of 100 s.

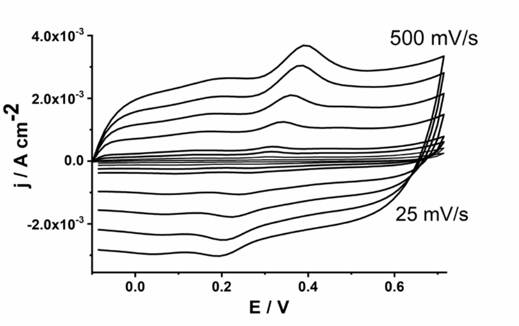

(**a**)

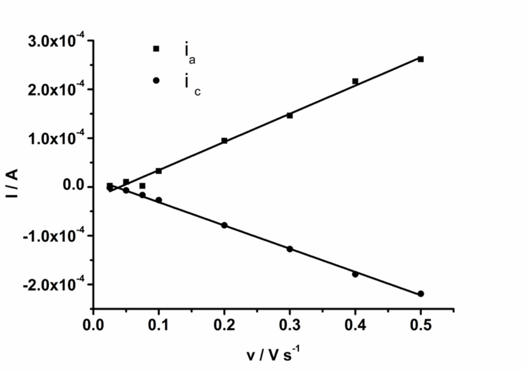

(**b**)

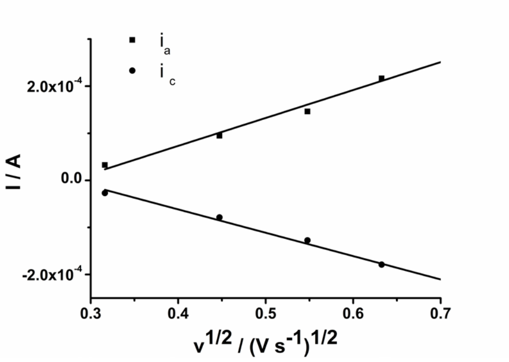

(**c**)

Figure S2. (**a**) CVs registered at PEDOT-AgNPs sensing platform in 0.1 M acetate buffer solution (pH = 5.0) containing 50 μM QR at various potential scanning rates from 25 to 500 mV/s. Anodic and cathodic peak-current dependence versus the (**b**) potential scan rate and (**c**) the square root of the potential scan rate for PEDOT-AgNPs sensing platform in 0.1 M acetate buffer containing 50 μM QR. Potential scan rate: 50 mV/s.

The corresponding linear regression equations for the peak-current dependence on the scan rate, and the dependence on the square root of the scan rate, respectively, were as follows:

I_a (A) = –2.3 × 10^{-5} + 5.8 × 10^{-4} v (V/s); r = 0.9961;

I_c (A) = 1.6 × 10^{-5} – 4.8 × 10^{-4} v (V/s); r = 0.9991;

I_a (A) = –1.6×10^{-4} + 5.8 × 10^{-4} $v^{1/2}$ $(V/s)^{1/2}$; r = 0.9940;

I_c (A) = 1.4×10^{-4} – 4.9 × 10^{-4} $v^{1/2}$ $(V/s)^{1/2}$; r = 0.9963.

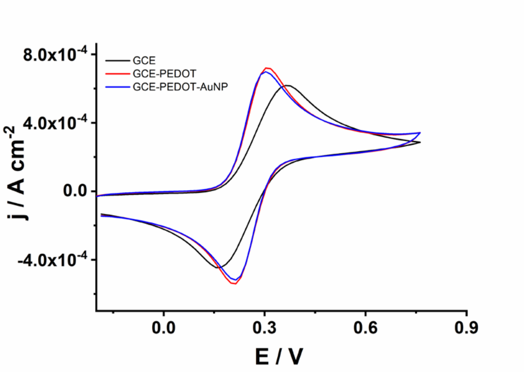

(**a**)

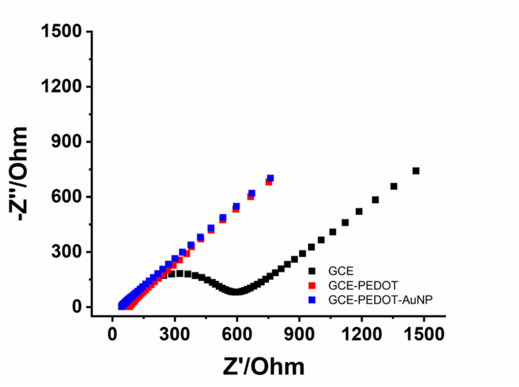

(**b**)

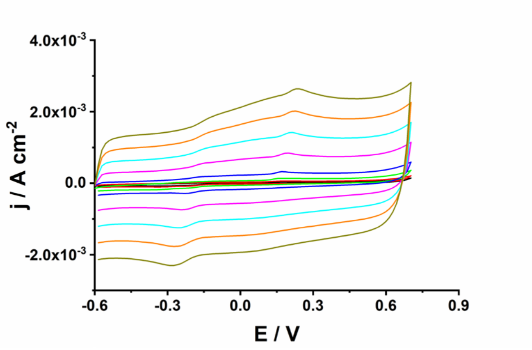

(c)

Figure S3. **(a)** CVs recorded in the presence of 5 mM $Na_4[Fe(CN)_6]$ at unmodified GCE, PEDOT-modified electrode, and PEDOT-AuNPs platform at 50 mV/s potential scanning rate. **(b)** EIS spectra for bare GCE, PEDOT, and PEDOT-AuNPs-modified electrodes in 5 mM $Na_4[Fe(CN)_6]$/ $K_3[Fe(CN)_6]$ solution at open circuit potential. **(c)** The analytical response of PEDOT-AuNPs sensing platform toward epinephrine in 0.1 M phosphate-buffered solution (pH = 7.0) containing 80 μM EPI at various potential scan rates: 10, 20, 50, 100, 200, 300, 400, and 500 mV/s.
